# The American Academy of Dental Surgery

**Published:** 1877-03

**Authors:** 


AMERICAN JOURNAL

OF

DENTAL SCIENCE.

Vol. X. THIRD SERIES—MARCH, 1877. No. 11.

### ARTICLE I.

### *The American Academy of Dental Surgery.*

[Reported for the American Journal of Dental Science.]

The regular meeting was held on the fourth Tuesday in January. Dr. George H. Perine, President, in the chair. The records of the last meeting read and approved. The reports of different committees were made and accepted. Donations to the library consisting of several volumes of medical and dental publications were received, and a vote of thanks returned.

Dr. Hayward read letters from corresponding Fellows, which were interesting and instructive.

The President stated that since the last meeting of the Academy, he was in receipt of letters requesting his theory, respecting the use of Anæsthetics for dental operations. After a few pertinent remarks upon the subject, he called for an expression from the Fellows present upon the subject.

Dr. Reed said that it was an important subject for the dentist to consider; he said there is a stage of the anæsthetic process, a stage especially well marked in robust moles, during which, the inhalation should be carried on slowly, for it is a stage of danger. You have all seen it doubtless, and must have marked how respiration is interfered with, slow jerking, or for a time entirely suspended. Over this stage always go slowly; remember the residual of respiration, the amount of air always in the lungs, not undergoing tidal movement. It may amount to as much as 250 cubic inches, which if saturated with chloroform vapour the temperature of 66° Fahr., would contain the vapour of 30 minims, or nearly twice as much as the average quantity present in the blood of an adult when sufficiently insensible for a surgical operation. That the air is not saturated, and generally not half saturated, is the reason why we do not hear of still more accidents. You will readily understand that this vapour of the residual air is being absorbed, even if respiration is entirely suspended, and now if you mark that at the close of this stage, the patient usually draws several deep inspirations, you can see how easy it may be to give an over dose. Keep in mind then the residual air of the lungs during the administration of chloroform, and especially during the stage of struggling.

Dr. Robertson said he thought there was often a want of sufficient care exercised in the administration of anæsthetics. He cosidered it unsafe to administer these agents before first understanding the physical condition of the patient receiving them; he fully concurred with Dr. Perine, that patients affected with apoplectic tendencies could never run the risk of inhaling an anæsthetic.

After some other remarks upon the subjects, Dr. Woodward said he regreted that he had not been present at the last meeting of the Academy, that he might have listened to Dr. Hart's paper, and the discussion on Neuralgia, a subject in which he was particularly interested. He would like the privelege of saying a few words upon the subject.

He would first draw attention to the use of chloral in Neuralgia. Liebreich, he said has shown that this drug is essentially an anæsthetic, and in large doses a hypnotic; that anæsthesia always begins in the head, and only after this region is fully under the influence of the croton-chloral and sleep has been induced, does the anæsthetic effect gradually extend over the rest of the body; with large doses the respiration and pulse become slower, and fatal result is due to arrest of the respiratory movements, not to the paralysis of the heart; in this respect croton-chloral differs from chloral, as with the latter death occurs directly from cesstion of the heart's action I have prescribed the drug for a number of patients with satisfaction. He gave a case of neuralgic headache. The pain at the beginning is generally limited to a small area of varying position, and is usually unilateral; it may remain localized, but more often spreads from that spot over a greater or less extent of surface, and may affect both sides, or be simply hemicranial. It is not necessarily limited to the region of any one nerve, but may at the height of the attack effect the whole scalp. The pain as is usual with neuralgia varies in character and intensity; after the pain has ceased, the scalp is often left very tender for a day or two, so that female patients will frequently say that the head is so sore that they cannot comb out the hair.

Migraine sick headache, or bilious headache, is an important variety. This is not the place to enter into a discussion of the pathology of this affection, but I may say that my experience has led me to adopt the opinion held by the late Dr. Anstie, that it is simply a neuralgia. Any how, it cannot be too distinctly remembered that it is a fine neurosis, and is not due to digestive disturbance. It is not an uncommon thing to meet with patients, particularly anæmic women, who, suffering from what they know as bilious sick-headache, fly for relief to so-called antibilious pills, and the like, which comes far from curing the malady that is the outcome of nervous exhaustion, and the element of digestive derangement, aggravate the original complaint.

In migraine, the act of vomiting is in fact, only an expression of the nervous exhaustion; and this act may be associated with severe neuralgias of other parts of the body, than the head. He gave the treatment of cases of the above varieties of neuralgia; for an adult the dose was five grains three times a day, some times relief was immediate.

Dr. J. C. Perine said as to the treatment of neuralgia as a matter of course, much depended he thought upon the cause of the affection. He gave a case: the patient had for months been suffering with very severe neuralgic pains in the face and head; at times the pain would go down to the sacrum, and hips; he was weak and ill, pulse low, appetite poor, was cured with belladonna administered three times a day. He said the profession would be interested in the work just published, (on the subject of neuralgia,) by Prof. Austin Flint.

Dr. Searl presented a case of facial neuralgia, which he thought shows the necessity to diagnosis a case before prescribing or operating. He said the patient had suffered much, and had all the lower molar teeth removed, in hopes of obtaining relief, yet it only produced a temporary cessation of pain; had taken many preparations, but none gave her a moment's freedom from pain, on examining her mouth found an abscess pointing over the cavity where the first molar had been extracted, and which was immediately opened; a quantity of fetid dark colored pus was discharged, and with it a somewhat pointed piece of salivary calculus. The pain iu her face ceased and from that time till the present, she has had no return of the neuralgia.

An interesting paper upon the "Tumors of the Mouth," by J. P. Buddington, M. D., was read. The subject was treated mainly from the stand point of personal experience. The author dwelt upon the best mode of treatment, both surgical, and medical; the removal of tumors—a consideration of the several agents which are supposed to control the growth of tumors.

Following the reading of this paper, (as the discussion was reserved for another meeting:)

C. P. Larich, M. D., Msocow, Russia, detailed some interesting cases in medical and dental practice, which excited considerable interest in the minds of his auditors. He paid a glowing tribute to American teaching and practice, and sketched the advances and discoveries, which has been made by Americans, and accepted by the profession in all parts of the world.

Some remarks followed, when the meeting adjourned to the fourth Tuesday in February.

